# A Case for Abandoning Inpatient Fecal Occult Blood Testing

**DOI:** 10.7759/cureus.8807

**Published:** 2020-06-24

**Authors:** Gregory T Brennan, Andrew S Parsons

**Affiliations:** 1 Gastroenterology, Texas Digestive Disease Consultants, Dallas, USA; 2 Internal Medicine, University of Virginia School of Medicine, Charlottesville, USA

**Keywords:** gastroenterology, high value care, endoscopy, choosing wisely, gi bleeding, fobt, occult blood

## Abstract

Fecal occult blood testing (FOBT) is currently Food and Drug Administration (FDA) approved only for colorectal cancer (CRC) screening. There is now widespread off-label use of FOBT in the hospital setting as a diagnostic test. Here we present a brief case and a more detailed review of the literature arguing against inpatient FOBT. Inpatient use of FOBT is problematic for several reasons including failure to account for false positives or negatives, delays in appropriate consultations or endoscopy, increased costs, increase length of stays, unnecessary procedures, and test results that do not change management. Inappropriate use of FOBT can lead to both overuse and underuse of endoscopy. Many retrospective audit studies and more recently a meta-analysis have shown that FOBTs have poor test performance and are unable rule out the need for endoscopy in patients with iron deficiency anemia. For these reasons we argue that inpatient FOBT should be abandoned.

## Introduction

Fecal occult blood testing (FOBT) can be done with guaiac FOBTs (gFOBT) or with fecal immunochemical tests (FIT). Guaiac based FOBT turns positive after a peroxidase reaction to the presence of heme. FIT detects human globin. Compared to FIT, gFOBTs are less accurate for the detection of colorectal neoplasia because heme is less specific than human globin. Both gFOBT and FIT are recommended screening tests for colorectal cancer (CRC) in the outpatient setting [1]. Although FOBTs are currently Food and Drug Administration (FDA) approved only for CRC screening, there is now widespread inappropriate use in the hospital setting as a diagnostic test [2]. Here we present a brief case and a discussion arguing against the utility of FOBT as a diagnostic test.

## Case presentation

A 56-year-old woman presented with painless, intermittent hematochezia. Her most recent episode of bleeding was two days earlier. Her past medical history included hypertension and hyperlipidemia. The patient was not taking aspirin, non-steroidal anti-inflammatory drugs (NSAIDs), or anticoagulation. She was afebrile with a heart rate of 101 beats/min, blood pressure of 105/70 mmHg, respiratory rate of 12 breaths/min, and oxygen saturation of 97%. The physical exam was normal. Laboratories were significant for a new microcytic anemia with a hemoglobin of 8.9 mg/dl. FOBT was performed on normal appearing stools and was negative. Even with the negative FOBT, endoscopy was pursued. Esophago-gastroduodenoscopy (EGD) and colonoscopy were negative for a culprit lesion. Video capsule endoscopy revealed fresh blood in the proximal small bowel and a push enteroscopy was ultimately done which revealed active bleeding from a distal duodenal submucosal gastrointestinal stromal tumor (GIST).

This case illustrates an example of how a false negative FOBT led to increased direct costs and no change in management.

## Discussion

Inpatient use of FOBT is problematic for several reasons. The test characteristics are accompanied by false positives and negatives, increased direct and indirect costs, delays in appropriate consultations or endoscopy, increase length of hospital stays, unnecessary procedures, and results that do not change management. Inappropriate use of FOBT can lead to both overuse and underuse of endoscopy [2-5]. 

False-positive FOBT results can occur via a variety of mechanisms including swallowed blood from nasopharyngeal or pulmonary sources, gastrointestinal (GI) inflammatory conditions (such as inflammatory bowel disease), medications (aspirin, NSAIDs), alcohol, ingestion of meats (which contains heme) and some fruits or vegetables containing peroxidase. False negatives can result from slow or intermittently bleeding lesions, proximal GI tract lesions, or high doses of vitamin C ingestion [4,6].

Several studies on the utility of inpatient gFOBT have found that the initial rationale for testing are also indications for endoscopy and FOBT results should not determine the need for endoscopy [2-5,7]. Uninvestigated iron deficiency anemia (IDA) and overt GI bleeding are both appropriate indications for endoscopy and are two of the most common reasons for obtaining a gFOBT in most audit studies. A negative gFOBT may lead to harm by delaying necessary investigations in this setting. One such case report in the literature detailed a patient with a negative gFOBT that had presented with melena and anemia [8]. The patient went on to have continued bleeding requiring transfusions and there was a delay in endoscopy because of a false negative gFOBT. Delays in endoscopy have led to prolonged hospital stays, increased costs, as well as harm to patients [3-5,9].

We know FOBTs are frequently done reflexively because audit studies have shown that positive gFOBTs are frequently ignored and appropriate investigations are never pursued [4,10]. In one such audit study, gFOBTs were done reflexively at the time of digital rectal exam (DRE) with no other indication for testing being identified [5]. Routine gFOBTs should not be done at the time of DRE. It is thought micro-tears during DRE could lead to false positives [11]. When done for colon cancer screening (as outpatient), stool samples should be collected after defecation. Because of false positive tests, patients will be referred for unnecessary endoscopy and therefore have exposure to unnecessary risk. One recent study found a large burden of inappropriate testing leading to unnecessary colonoscopies with an estimated rate of only one case of CRC diagnosed for every 214 patients inappropriately tested with FOBT [2]. 

Inappropriate fecal occult blood testing is most commonly obtained to evaluate for occult GI bleeding in patients with anemia [3,9]. In patients with occult GI bleeding, one would expect to find IDA. Uninvestigated iron deficiency without an obvious cause warrants an endoscopic evaluation regardless of the results of the FOBT. The belief that FOBT is useful at picking up lesions in the upper GI tract is wrong. Patients with a positive FIT and a negative colonoscopy rarely have an upper GI malignancy [12]. Human globin is rapidly degraded during transit through the proximal gastrointestinal tract and consequently FIT has a low sensitivity for proximal bleeding lesions [13]. After analyzing the Netherlands national cancer registry of over 16,000 patients, van der Vlugt et al. found no difference in the incidence of oral, throat, esophageal, or gastric cancers between FIT positives and FIT negatives [12]. In patients with a positive FOBT and a negative colonoscopy, routine endoscopy is not recommended because the number needed to scope to find a significant lesion is not cost effective [12].

The strongest evidence to date arguing against the use of FOBT as a diagnostic test for anemia was recently published in 2020 [14]. This systematic review and meta-analysis found that a negative FOBT does not rule out the need for endoscopy in patients with IDA because of a high false negative rate. The authors found that the sensitivity of FOBT was low (58%) in IDA. Furthermore, 42% of patients with negative tests were found to have endoscopically identifiable causes of IDA. These results were similar in both guaiac-based testing and FIT. In addition, they found that FOBTs are not useful in identifying upper gastrointestinal tract malignancy. Ten of the 18 cases of upper gastrointestinal malignancy (esophageal, gastric, and proximal small bowel) had a negative FOBT. In addition, the utility of FOBT for patients with acute diarrhea and ulcerative colitis were analyzed with inconclusive results. The authors concluded that physicians should not use the results of FOBTs to guide decisions regarding the need for endoscopic evaluations [14].

## Conclusions

Inpatient testing of FOBT should be eliminated by hospitals in an effort to reduce costs and prevent harm to patients. Visual characterization of the stool is the most helpful information to determine the need for endoscopy. In patients with melena or hematochezia, a FOBT should be abandoned to prevent delays in endoscopy. FOBTs perform poorly as a diagnostic test and play no role in the work up for occult GI bleeding in any setting. A negative FOBT has poor test performance and is unable rule out the need for endoscopy in patients with IDA. Furthermore, asymptomatic patients with a positive FOBT and a negative colonoscopy do not need an EGD (in the absence of IDA or upper GI symptoms). A focused history, physical exam, and laboratory investigations should lead to a diagnosis of GI hemorrhage and trigger appropriate endoscopic evaluations without the need for FOBT testing. For these reasons, we argue that inpatient FOBT should be abandoned.

## References

[1] Wolf AMD, Fontham ETH, Church TR (2018). Colorectal cancer screening for average-risk adults: 2018 guideline update from the American Cancer Society. CA Cancer J Clin.

[2] Soin S, Akanbi O, Ahmed A, Kim Y, Pandit S, Wroblewski I, Saleem N (2019). Use and abuse of fecal occult blood tests: a community hospital experience. BMC Gastroenterol.

[3] Ip S, Sokoro AAH, Kaita L, Ruiz C, McIntyre E, Singh H (2014). Use of fecal occult blood testing in hospitalized patients: results of an audit. Can J Gastroenterol Hepatol.

[4] Narula N, Ulic D, Al-Dabbagh R, Ibrahim A, Mansour M, Balion C, Marshall JK (2014). Fecal occult blood testing as a diagnostic test in symptomatic patients is not useful: a retrospective chart review. Can J Gastroenterol Hepatol.

[5] Gupta A, Tang Z, Agrawal D (2018). Eliminating in-hospital fecal occult blood testing: our experience with disinvestment. Am J Med.

[6] Mathews B, Ratcliffe T, Sehgal R, Abraham JM, Monash B (2017). Fecal occult blood testing in hospitalized patients with upper gastrointestinal bleeding. J Hosp Med.

[7] Mosadeghi S, Ren H, Catungal J, Yen I, Liu B, Wong RJ, Bhuket T (2016). Utilization of fecal occult blood test in the acute hospital setting and its impact on clinical management and outcomes. J Postgrad Med.

[8] Bangaru S, Tang D, Agrawal D (2018). Inappropriate use of fecal occult blood testing. JAMA Intern Med.

[9] Friedman A, Chan A, Chin L (2010). Use and abuse of faecal occult blood tests in an acute hospital inpatient setting. Intern Med J.

[10] Sharma VK, Komanduri S, Nayyar S (2001). An audit of the utility of in-patient fecal occult blood testing. Am J Gasternetol.

[11] Ransohoff D, Lang C (1997). Screening for colorectal cancer with the fecal occult blood test: a background paper. Ann Intern Med.

[12] van der Vlugt M, Grobbee EJ, Bossuyt PM (2018). Risk of oral and upper gastrointestinal cancers in persons with positive results from a fecal immunochemical test in a colorectal cancer screening program. Clin Gastroenterol Hepatol.

[13] Young GP, St John DJ, Rose IS (1990). Haem in the gut. Part II. Fecal excretion of haem and haem-derived porphyrins and their detection. J Gastroenterol Hepatol.

[14] Lee MW, Pourmorady J, Laine L (2020). Use of fecal occult blood testing as a diagnostic tool for clinical indications. Am J Gastroenterol.

